# Investigation of the effects of T-2 toxin in chicken-derived three-dimensional hepatic cell cultures

**DOI:** 10.1038/s41598-024-51689-1

**Published:** 2024-01-12

**Authors:** Júlia Vörösházi, Máté Mackei, Csilla Sebők, Patrik Tráj, Rege Anna Márton, Dávid Géza Horváth, Korinna Huber, Zsuzsanna Neogrády, Gábor Mátis

**Affiliations:** 1 Division of Biochemistry, Department of Physiology and Biochemistry, University of Veterinary Medicine, Budapest, 1078 Hungary; 2 National Laboratory of Infectious Animal Diseases, Antimicrobial Resistance, Veterinary Public Health and Food Chain Safety, University of Veterinary Medicine, Budapest, 1078 Hungary; 3 Department of Pathology, University of Veterinary Medicine, Budapest, 1078 Hungary; 4 Institute of Animal Science, University of Hohenheim, 70599 Stuttgart, Germany

**Keywords:** Cell biology, Molecular biology

## Abstract

Despite being one of the most common contaminants of poultry feed, the molecular effects of T-2 toxin on the liver of the exposed animals are still not fully elucidated. To gain more accurate understanding, the effects of T-2 toxin were investigated in the present study in chicken-derived three-dimensional (3D) primary hepatic cell cultures. 3D spheroids were treated with three concentrations (100, 500, 1000 nM) of T-2 toxin for 24 h. Cellular metabolic activity declined in all treated groups as reflected by the Cell Counting Kit-8 assay, while extracellular lactate dehydrogenase activity was increased after 500 nM T-2 toxin exposure. The levels of oxidative stress markers malondialdehyde and protein carbonyl were reduced by the toxin, suggesting effective antioxidant compensatory mechanisms of the liver. Concerning the pro-inflammatory cytokines, IL-6 concentration was decreased, while IL-8 concentration was increased by 100 nM T-2 toxin exposure, indicating the multifaceted immunomodulatory action of the toxin. Further, the metabolic profile of hepatic spheroids was also modulated, confirming the altered lipid and amino acid metabolism of toxin-exposed liver cells. Based on these results, T-2 toxin affected cell viability, hepatocellular metabolism and inflammatory response, likely carried out its toxic effects by affecting the oxidative homeostasis of the cells.

## Introduction

Mycotoxins are secondary metabolites produced by different species of fungi that can cause agricultural damage worldwide by contaminating feed^1^. Among others, the most prominent mycotoxins are the trichothecenes produced by various *Fusarium*, *Myrothecium* and *Stachybotrys* species. One important member of the trichothecenes is the T-2 toxin originated from different *Fusarium* species, including *F. soprotrichioides*, *F. poae*, and *F. acuinatum*, that contaminate a wide range of cereals and are most prevalent in cold climates or under wet storage conditions^1^. The most commonly affected cereals are maize, wheat and oat, which are highly involved in both human nutrition and livestock feeding^2^. The uptake of T-2 toxin happens by food or water intake, by inhalation of air or aerosols and by transdermal absorption. Most of the T-2 toxin is absorbed from the gastrointestinal tract and subsequently transported to the liver via the *v. portae*, which is thereby directly exposed to toxic compounds of enteric origin^1,3,4^.

Poultry species as mainly grain consumers may come into regular contact with mycotoxins, including T-2 toxin which can be accumulated in their feed due to inadequate plant production, harvesting or storage conditions^5^. They are generally more tolerant to trichothecenes than mammalian species^6^. The lower susceptibility is probably due to the moderate absorption as well as the rapid metabolism and elimination of these mycotoxins. On the other hand, exposure to these mycotoxins has serious negative effects in these animals too, and is a major problem for the poultry industry worldwide^6,7^. The major detrimental effects of T-2 toxin in poultry include reduced growth and appetite, modified immune response, gastrointestinal impairment, neurological and reproductive disorders, mostly mediated by the inhibition of protein synthesis on the cellular level^8,9^. Furthermore, T-2 toxin is known to induce apoptosis, and it may also elevate free radical production causing oxidative stress, which is associated with DNA damage and increased lipid peroxidation^1,6^. Certain signalling pathways that are strongly linked to reactive oxygen species (ROS) responses, such as the nuclear factor (erythroid-derived 2)-like 2/heme oxygenase 1 (Nrf2/HO-1) pathway, the endoplasmic reticulum (ER) stress pathway, and the mammalian target of rapamycin/protein kinase (mTOR/Akt) system, appear to be involved in the toxicity caused by T-2 toxin^10^. In addition, T-2 toxin stimulated the gene expression of specific proinflammatory cytokines, such as interleukin- (IL-)1α, IL-1β, IL-6, IL-11 and tumor necrosis factor-α (TNF-α) both in vitro and in vivo^11^.

The possible oxidative stress caused by the toxin is often associated with the development of ER stress^12,13^. In physiological conditions, newly synthesized proteins are folded and modified in the ER. These processes are controlled by various chaperones and folding enzymes such as glucose-regulated protein 78 (GRP78), a member of the heat shock protein 70 (HSP70) family, located in the membrane of the ER. If the folding of the produced proteins is not sufficient due to various stress factors, the ER triggers the unfolded-protein response (UPR) mechanism to restore the cell homeostasis or, in severe cases, to induce apoptosis^14^.

Oxidative and ER stress also affect the production of certain small heat shock proteins (sHSPs). These proteins have a molecular mass of about 15–30 kDa and are often involved in response to various stress factors. Among them, early reversible phosphorylation of heat shock protein 27 (HSP27) upon oxidative and ER stress has been reported in several cell types^13,15^. If the protein homeostasis is not restored after oxidative or ER stress, cell and tissue damage occurs. In response, inflammatory processes are triggered, which limit the extent of tissue damage and promote tissue regeneration. Several studies have already reported the complex interactions between oxidative stress, ER stress and inflammation, although the exact interplay of these processes are not yet known^13,14^.

The majority of in vitro and in vivo studies on T-2 toxin have focused mainly on a single metabolic pathway or organ in one experiment, using conventional biochemical and molecular biology methods^11,16,17^. To further understand the toxic effects of this molecule, a comprehensive study of the systemic metabolic effects of T-2 toxin was required^18^. Metabolomics as a novel and increasingly widely used approach in life sciences can provide a coherent overview of the metabolic subsystems of the organism, rather than studying a few components at a time^19^.

Hepatic cell cultures can serve as proper models for studying the cellular effects of T-2 toxin on the liver as a major site of mycotoxin exposure. In recent years, three-dimensional (3D) cell cultures have become widely used due to their numerous advantages over traditional two-dimensional (2D) cell cultures. In monolayer cultures, hepatic cells are prone to dedifferentiation and loss of morphology, probably due to the limited contact between cells and between cells and the extracellular matrix (ECM)^20^. In contrast, 3D cell cultures are able to develop a microenvironment and function similar to the physiological conditions^21^. In these cultures, cells keep their phenotype and characteristic functions as well as their ability to grow and interact with their surroundings, allowing the formation of diverse cell–cell and cell–ECM interactions^22^. In this study, magnetic 3D bioprinting was used to create 3D hepatic cell cultures. By this method, isolated cells are incubated with magnetic nanoparticles consisting of iron oxide, gold, and poly-l-lysine. These nanoparticles electrostatically bind to the cell membranes via the poly-l-lysine at a concentration of approximately 50 pg/cell. At this rate, the nanoparticles coat the cells in a scattered manner rather than the entire membrane, giving them a pepped-like appearance^23,24^. However, this amount is enough to magnetize the cells, therefore they are able to aggregate into spheroids when placed in magnetic field^20^. The nanoparticles are biocompatible and have no effect on the cellular viability and proliferation^25–27^. Additionally, Tseng et al. also observed that their effect on inflammatory processes was minimal and negligible in 3D multitype bronchiole co-cultures^26^. Furthermore, it was also demonstrated in aortic valve 3D co-cultures that they had no significant effect on the cellular oxidative processes^27^.

The aim of this study was to investigate the cellular effects of T-2 toxin on oxidative and ER stress as well as inflammatory response in the chicken liver, carried out on a newly characterized chicken derived primary 3D hepatic cell culture gained by magnetic bioprinting. As 3D cell cultures may reflect in vivo conditions more accurately than traditional monolayer cultures, the applied 3D cell culture can serve as a good model to monitor the effects of mycotoxins on the avian liver. The present study can also provide an insight into the complex interplay of the ER and oxidative stress with the inflammatory pathways. Moreover, to investigate the cellular effects of T-2 toxin, a wide range of metabolites related to amino acid, glucose and lipid metabolism as well as to inflammatory processes were assessed by a targeted metabolomics approach.

## Results

### Haematoxylin and eosin (H&E) staining

**Figure 1:**
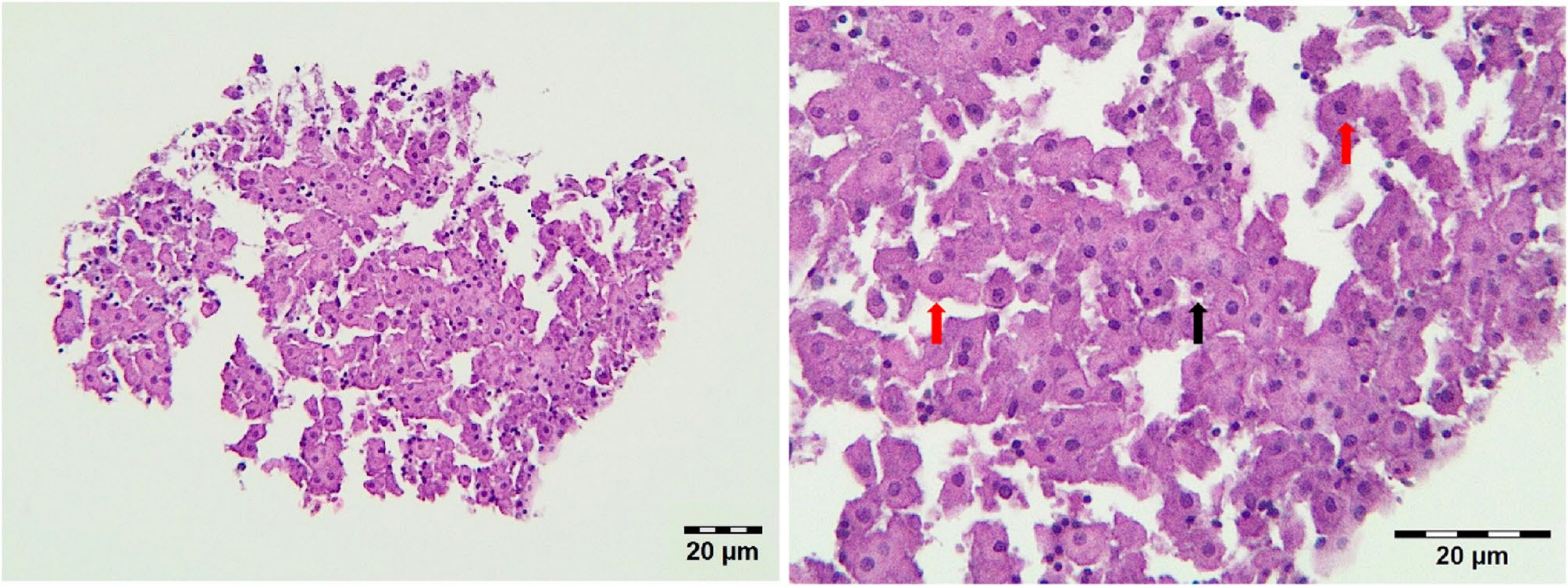
Primary 3D hepatic spheroids from chicken origin. The black arrow shows an NP cell, while red arrows show hepatocytes. H&E staining, bar: 20 µm.

Figure 1 shows a formalin fixed paraffin embedded (FFPE) slice after haematoxylin and eosin (H&E) staining. Morphologically intact hepatocytes are observable in high abundance in loose connection with each other as well as other non-parenchymal (NP) cells with smaller nucleus. Severe degenerative changes are not visible.

### Metabolic activity

**Figure 2:**
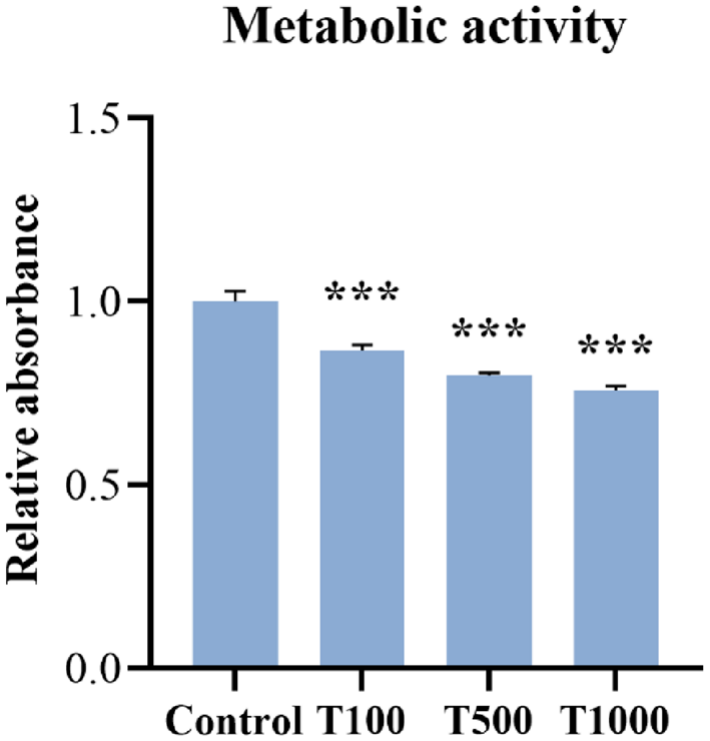
Effects of 24 h T-2 toxin treatment on the metabolic activity of primary hepatic 3D cell co-cultures of chicken origin assessed by CCK-8 test. Control: cells without T-2 toxin exposure; T100: 100 nM, T500: 500 nM, T1000: 1000 nM T-2 toxin treatment. Relative absorbances were calculated by considering the mean value of the Control group as 1. Results are expressed as mean ± SEM. ***p < 0.001.

Metabolic activity of the cultured cells was measured by Cell Counting Kit-8 (CCK-8) test from the cell culture media after treatment with 100, 500 and 1000 nM T-2 toxin for 24 h. T-2 toxin significantly decreased (*p* < 0.001) the metabolic activity in every treatment group compared to the control (Fig. 2). The mean ± SEM values obtained from the CCK-8 measurement are shown in Supplementary Table 1.

### Lactate dehydrogenase (LDH) activity

**Figure 3:**
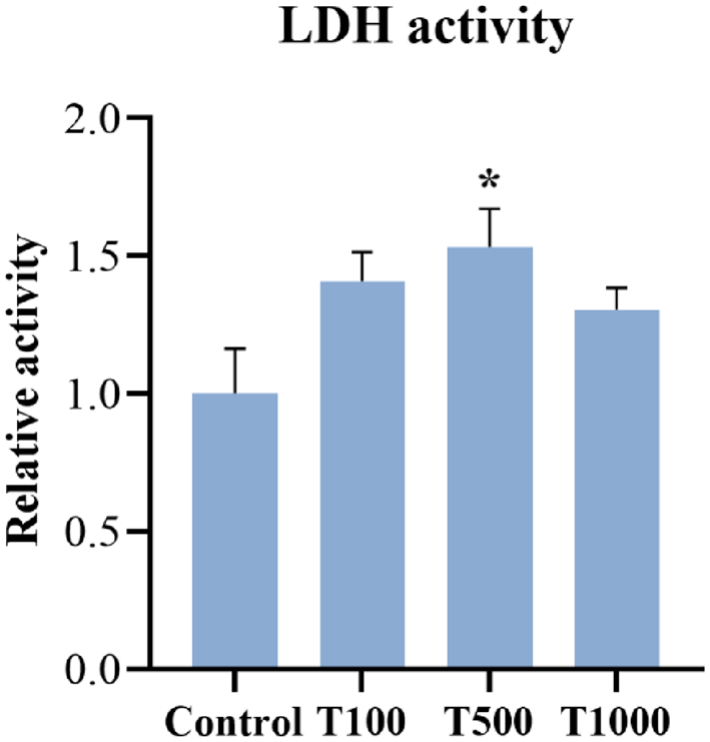
Effects of 24 h T-2 toxin treatment on the LDH activity of primary hepatic 3D cell co-cultures of chicken origin. Control: cells without T-2 toxin exposure; T100: 100 nM, T500: 500 nM, T1000: 1000 nM T-2 toxin treatment. Relative activity was calculated by considering the mean value of the Control group as 1. Results are expressed as mean ± SEM. *p < 0.05.

The extracellular LDH activity of the cell cultures was measured after 24 h of T-2 toxin treatment. The 500 nM T-2 toxin concentration significantly increased (*p* = 0.038) the LDH activity of the cells in comparison with the control group (Fig. 3). The mean ± SEM values obtained from the LDH activity measurement are shown in Supplementary Table 1.

### Malondialdehyde (MDA) and protein carbonyl (PC) concentrations

**Figure 4:**
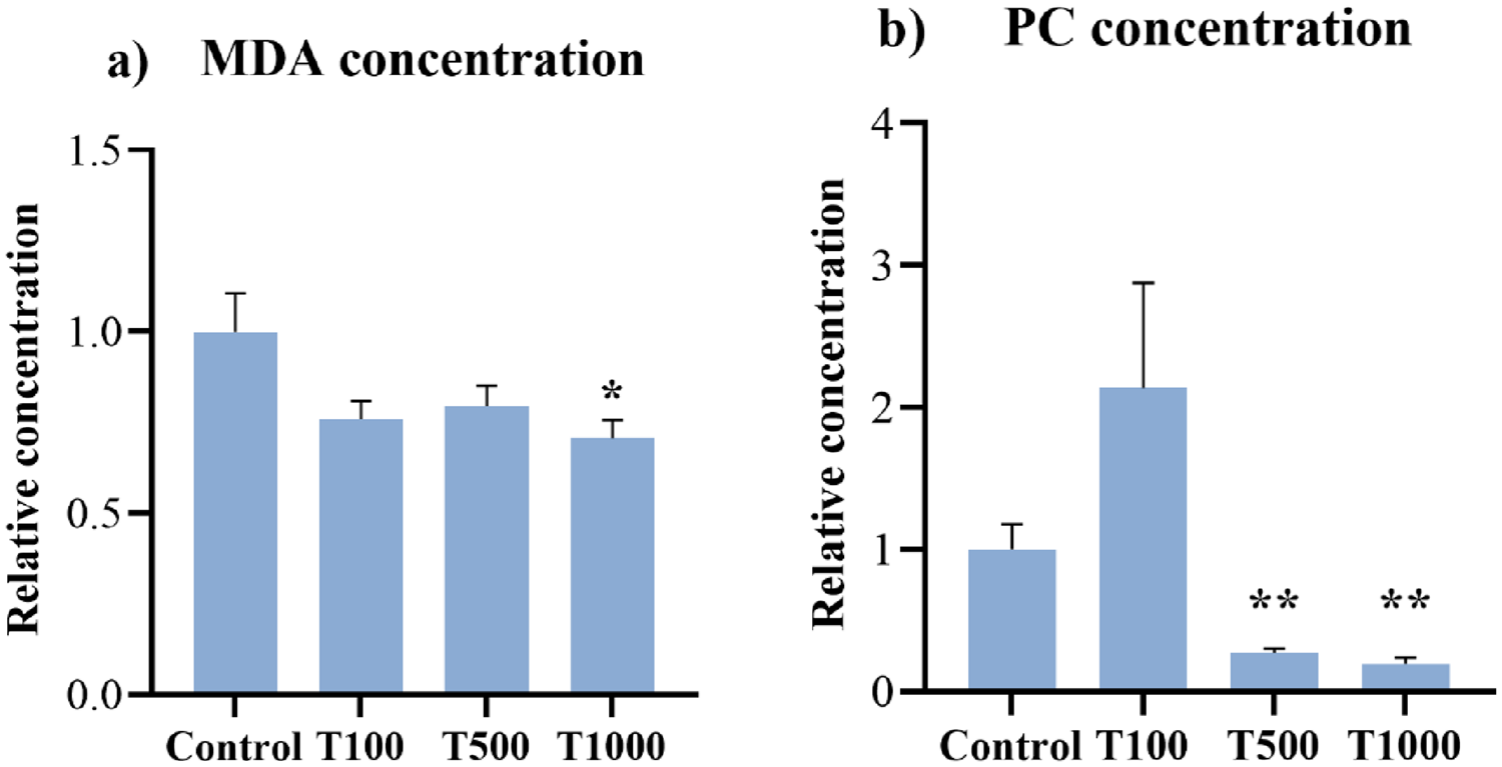
Effects of 24 h T-2 toxin treatment on the (**a**) MDA and (**b**) PC concentrations of primary hepatic 3D cell co-cultures of chicken origin assessed by chicken specific ELISA test. Control: cells without T-2 toxin exposure; T100: 100 nM, T500: 500 nM, T1000: 1000 nM T-2 toxin treatment. Relative concentrations were calculated by considering the mean value of the Control group as 1. Results are expressed as mean ± SEM. *p < 0.05, **p < 0.01.

The MDA concentration was measured from the cell culture media and the PC content was determined from the cell lysates by chicken specific ELISA test after 24 h of T-2 toxin treatment. The MDA level was significantly decreased (*p* = 0.030) in the 1000 nM treatment group after 24 h (Fig. 4a). The higher levels of T-2 toxin (500 nM and 1000 nM) significantly decreased (*p* = 0.007, *p* = 0.001, respectively) the intracellular PC concentration of the cultured cells compared to the control group (Fig. 4b). The mean ± SEM values obtained from the MDA and PC measurements are shown in Supplementary Table 1.

### Interleukin (IL) concentrations

**Figure 5:**
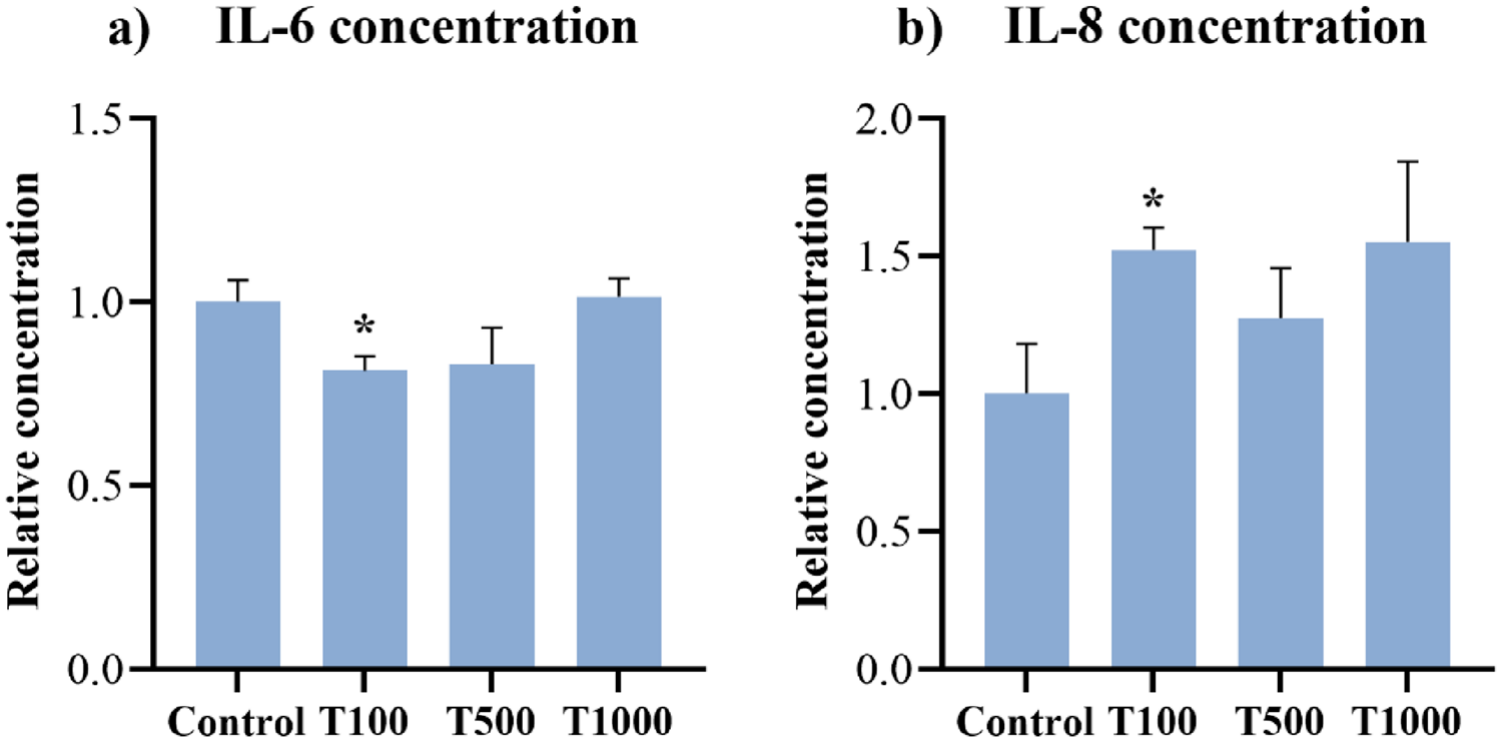
Effects of 24 h T-2 toxin treatment on the (**a**) IL-6 and (**b**) IL-8 concentrations of primary hepatic 3D cell co-cultures of chicken origin assessed by chicken specific ELISA test. Control: cells without T-2 toxin exposure; T100: 100 nM, T500: 500 nM, T1000: 1000 nM T-2 toxin treatment. Relative concentrations were calculated by considering the mean value of the Control group as 1. Results are expressed as mean ± SEM. *p < 0.05.

After 24 h of T-2 toxin treatment, the concentrations of IL-6 and IL-8 were assessed from the cell culture media using chicken-specific ELISA tests. The extracellular concentration of IL-6 was significantly lowered (*p* = 0.026) after 24 h by the 100 nM T-2 toxin treatment when compared to that of the control cells (Fig. 5a). The IL-8 concentration was significantly increased (*p* = 0.025) after 24 h in the 100 nM T-2 toxin treatment group (Fig. 5b). The mean ± SEM values obtained from the IL-6 and IL-8 measurements are shown in Supplementary Table 1.

### Glucose-regulated protein 78 (GRP78) and heat shock protein 27 (HSP27) concentrations

**Figure 6:**
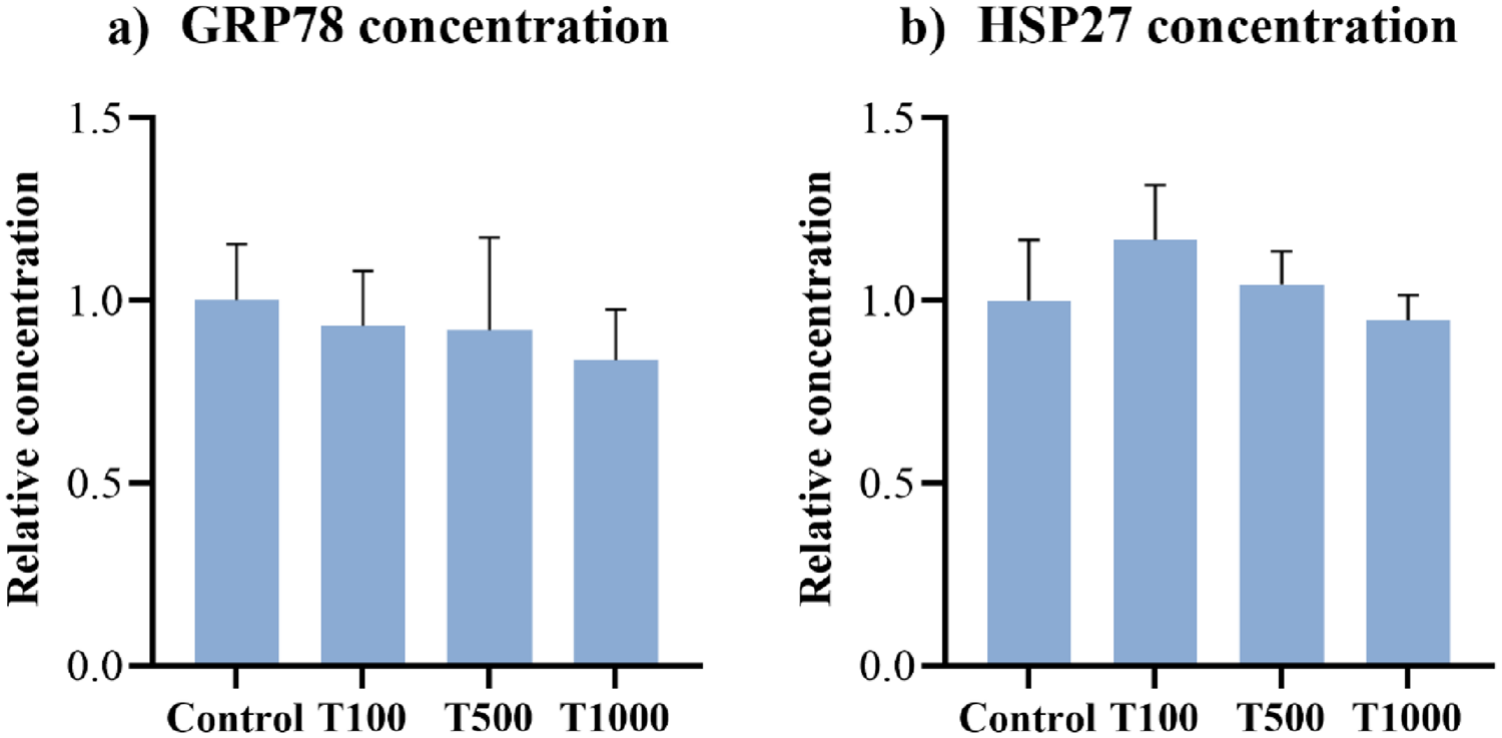
Effects of 24 h T-2 toxin treatment on the (**a**) GRP78 and (**b**) HSP27 concentrations of primary hepatic 3D cell co-cultures of chicken origin assessed by chicken specific ELISA test. Control: cells without T-2 toxin exposure; T100: 100 nM, T500: 500 nM, T1000: 1000 nM T-2 toxin treatment. Relative concentrations were calculated by considering the mean value of the Control group as 1. Results are expressed as mean ± SEM.

The GRP78 and HSP27 contents were assessed after 24 h of T-2 toxin treatment by chicken specific ELISA tests. The applied toxin exposures did not influence significantly the concentrations of these two parameters (Fig. 6a,b). The mean ± SEM values obtained from the GRP78 and HSP27 measurements are shown in Supplementary Table 1.

### Metabolome analysis

**Figure 7:**
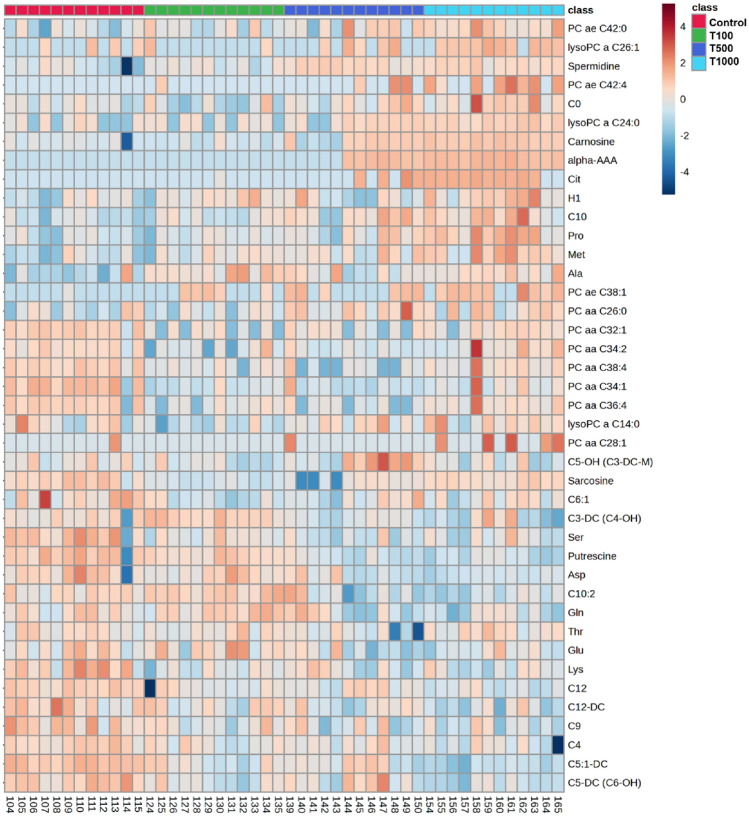
Heatmap visualising the concentrations of 41 metabolites in spheroid media with significantly changed abundance in response to 24 h T-2 toxin treatment. Control: cells without T-2 toxin exposure; T100: 100 nM, T500: 500 nM, T1000: 1000 nM T-2 toxin treatment.

Figure 7 and Table 1 show overviews of the 41 metabolites being significantly influenced by T-2 toxin exposure, visualised on a heatmap. As reflected by the colour scheme of the heatmap, most of the significantly changed metabolites showed T-2 toxin triggered increased abundance (alpha-aminoadipic acid [alpha-AAA], citrulline [Cit], proline [Pro], carnosine, spermidine, alanine [Ala], methionine [Met], decanoylcarnitine [C10], free carnitine [C0], hydroxyisovalerylcarnitine [C5-OH], phosphatidylcholine with acyl-alkyl residue sum [PC ae] C42:4, C38:1 and 42:0, phosphatidylcholine with diacyl residue sum [PC aa] C26:1 and C28:1, lysophosphatidylcholine with acyl residue [lysoPC a] C24:0 and 26:1, hexose [H1]), while the concentrations of other metabolites (glutamine [Gln], lysine [Lys], serine [Ser], threonine [Thr], aspartate [Asp], glutamate [Glug, putrescine, sarcosine, glutaconylcarnitine [C5:1-DC], decadienoylcarnitine [C10:2], glutarylcarnitine [C5-DC], hydroxybutyrylcarnitine [C3-DC], dodecanoylcarnitine [C12-DC], C12, nonanoylcarnitine [C9], hexenoylcarnitine [C6:1], butyrylcarnitine [C4], PC aa C34:1, PC aa C38:4, PC aa C36:4, PC aa C32:1, PC aa C34:2, lysoPC a C14:0) were found to be decreased after the T-2 toxin treatment compared to the control group.

**Table 1:** Metabolites significantly different from the control group. Statistics were calculated by ANOVA, pairwise comparisons by Tukey's HSD.

Metabolite	Control mean ± SEM	T100 mean ± SEM	T500 mean ± SEM	T1000 mean ± SEM	Significance
Alpha-AAA	ND	ND	0.323 ± 0.083	0.593 ± 0.022	***
Cit	ND	0.002 ± 0.002	0.664 ± 0.393	0.972 ± 0.140	***
Pro	281.750 ± 6.075	289.917 ± 5.032	302.750 ± 8.567	340.750 ± 8.816	***
Carnosine	0.196 ± 0.019	0.193 ± 0.012	0.321 ± 0.039	0.432 ± 0.008	***
Spermidine	0.094 ± 0.014	0.122 ± 0.009	0.199 ± 0.011	0.200 ± 0.009	***
Ala	937.583 ± 18.555	1034.750 ± 13.268	993.667 ± 16.078	1024.500 ± 9.882	***
Met	95.842 ± 2.056	101.833 ± 1.872	104.000 ± 2.086	111.117 ± 2.564	***
C10	0.634 ± 0.014	0.666 ± 0.014	0.726 ± 0.025	0.764 ± 0.025	***
PC ae C42:4	ND	ND	0.003 ± 0.002	0.007 ± 0.003	***
C0	1.095 ± 0.021	1.047 ± 0.041	1.173 ± 0.033	1.265 ± 0.052	***
PC ae C38:1	ND	0.004 ± 0.002	0.007 ± 0.002	0.018 ± 0.009	**
lysoPC a C24:0	0.040 ± 0.011	0.043 ± 0.009	0.079 ± 0.012	0.111 ± 0.006	**
PC ae C42:0	0.170 ± 0.003	0.176 ± 0.003	0.183 ± 0.005	0.191 ± 0.003	**
lysoPC a C26:1	0.013 ± 0.006	0.008 ± 0.005	0.023 ± 0.009	0.046 ± 0.007	**
H1	12,322.083 ± 240.933	13,085 ± 252.481	12,549.420 ± 323.376	12,753.250 ± 289.951	**
PC aa C26:0	0.288 ± 0.006	0.286 ± 0.005	0.313 ± 0.007	0.305 ± 0.006	**
C5-OH	0.073 ± 0.002	0.066 ± 0.003	0.086 ± 0.006	0.068 ± 0.003	**
PC aa C28:1	0.001 ± 0.001	ND	0.002 ± 0.002	0.010 ± 0.004	**
C5:1-DC	0.211 ± 0.006	0.142 ± 0.010	0.148 ± 0.011	0.101 ± 0.005	***
Putrescine	0.144 ± 0.012	0.128 ± 0.005	0.082 ± 0.005	0.076 ± 0.005	***
C10:2	0.200 ± 0.006	0.219 ± 0.006	0.166 ± 0.013	0.155 ± 0.006	***
C5-DC	0.133 ± 0.005	0.099 ± 0.005	0.115 ± 0.009	0.088 ± 0.004	***
PC aa C38:4	0.068 ± 0.006	0.028 ± 0.004	0.021 ± 0.008	0.074 ± 0.032	***
PC aa C34:1	0.219 ± 0.020	0.083 ± 0.008	0.089 ± 0.018	0.148 ± 0.043	***
Sarcosine	0.781 ± 0.069	0.316 ± 0.015	0.373 ± 0.069	0.685 ± 0.035	***
PC aa C36:4	0.068 ± 0.007	0.023 ± 0.004	0.017 ± 0.005	0.091 ± 0.052	***
Lys	535.250 ± 15.572	466.833 ± 9.482	473.833 ± 12.696	469.000 ± 10.462	***
C3-DC	0.152 ± 0.011	0.189 ± 0.008	0.122 ± 0.005	0.125 ± 0.019	**
Ser	136.633 ± 4.786	122.833 ± 2.522	115.333 ± 2.986	118.833 ± 3.353	**
Gln	2324.167 ± 35.918	2397.500 ± 33.803	2206.667 ± 62.284	2147.5000 ± 43.920	**
PC aa C32:1	0.035 ± 0.004	0.010 ± 0.003	0.013 ± 0.003	0.020 ± 0.003	**
C12-DC	0.465 ± 0.010	0.442 ± 0.009	0.430 ± 0.008	0.412 ± 0.010	**
C9	0.085 ± 0.003	0.074 ± 0.002	0.072 ± 0.003	0.069 ± 0.003	**
C12	0.075 ± 0.002	0.051 ± 0.005	0.058 ± 0.004	0.047 ± 0.003	**
PC aa C34:2	0.073 ± 0.006	0.040 ± 0.013	0.036 ± 0.005	0.177 ± 0.108	**
lysoPC a C14:0	0.253 ± 0.009	0.226 ± 0.006	0.255 ± 0.007	0.261 ± 0.008	**
C6:1	0.108 ± 0.006	0.087 ± 0.003	0.093 ± 0.004	0.094 ± 0.003	**
Thr	375.750 ± 5.015	372.917 ± 4.864	344.667 ± 11.981	377.250 ± 5.021	**
Asp	497.833 ± 28.112	489.333 ± 15.234	434.500 ± 17.047	409.083 ± 5.864	**
C4	0.068 ± 0.003	0.053 ± 0.004	0.053 ± 0.003	0.044 ± 0.005	**
Glu	360.750 ± 4.910	366.250 ± 6.426	341.500 ± 6.161	348.333 ± 4.552	**

ND: not detected under detection level.

Control: cells without T-2 toxin exposure; T100: 100 nM, T500: 500 nM, T1000: 1000 nM T-2 toxin treatment.

*p < 0.05, **p < 0.01, ***< 0.001.

Further, the metabolic profile of the control group was also compared to that of the cell-free culture medium. The list of metabolites significantly changed between the cell-free medium and the control group and the corresponding p-values are given in Supplementary Table 2. Overall, 72 metabolites changed significantly, of which 40 decreased and 32 increased in the control samples compared to the cell-free medium.

**Figure 8:**
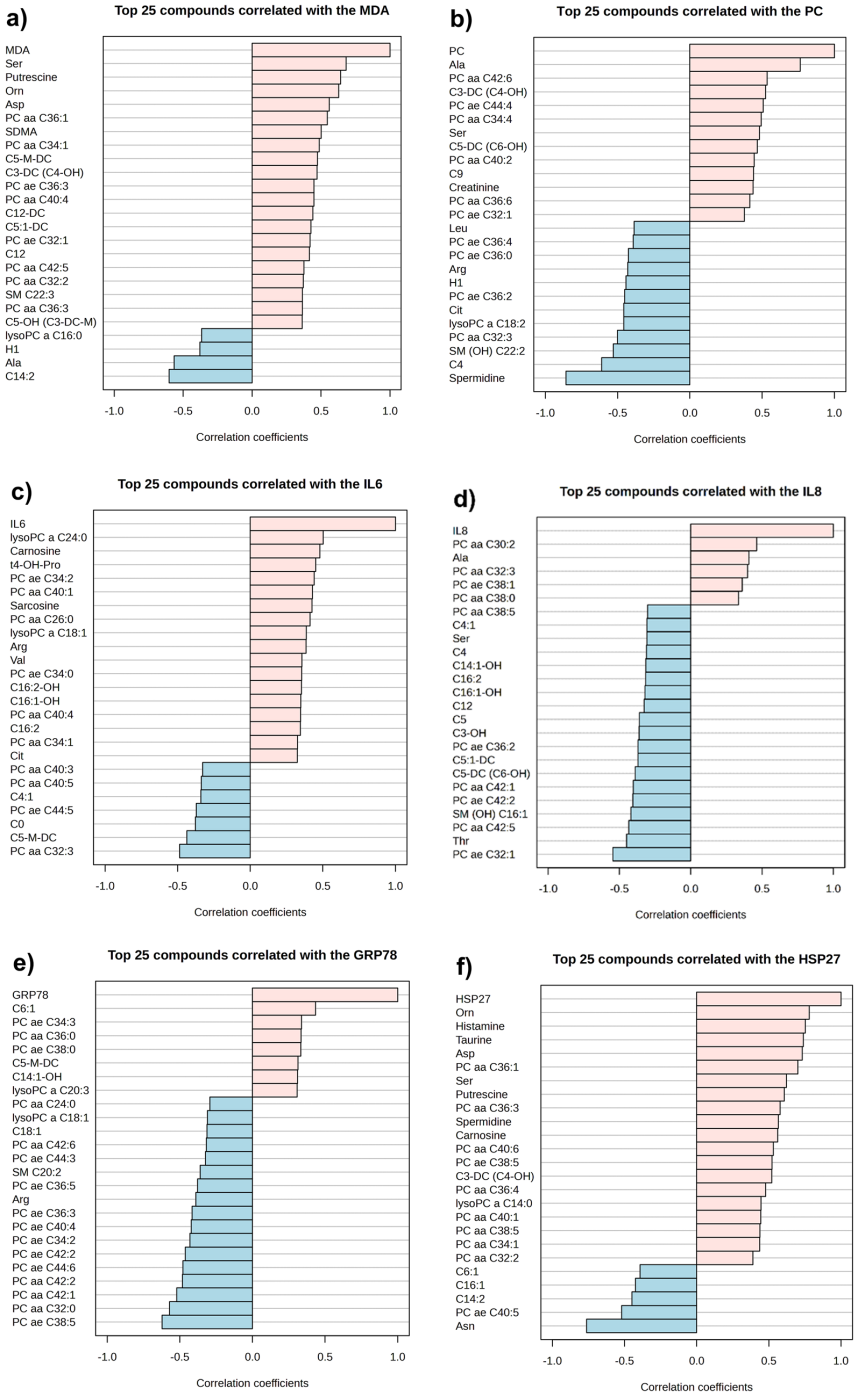
Correlation between the parameters measured by chicken-specific ELISA tests and metabolites measured by the AbsoluteIDQ p180 kit. Correlation between (**a**) MDA and metabolites, (**b**) PC and metabolites, (**c**) IL-6 and metabolites, (**d**) IL-8 and metabolites, (**e**) GRP78 and metabolites, (**f**) HSP27 and metabolites. Correlation was assessed using the Pattern Hunter function of the MetaboAnalyst 5.0 program.

Moderate (0.50 > R^2^ > 0.70) and high (0.70 > R^2^ > 0.90) positive as well as moderate (− 0.50 > R^2^ > − 0.70) and high (− 0.70 > R^2^ > − 0.90) negative correlations were found between the concentrations of many metabolites and the parameters (MDA, PC, IL-6, IL-8, GRP78 and HSP27) determined by chicken-specific ELISA tests. These correlations are depicted in Fig. 8a–f.

Three amino acids (ornithine [Orn], Ser, Asp), two biogenic amines (putrescine, symmetric dimethylarginine [SDMA]) and a phosphatidylcholine (PC aa C36:1) were correlated positively (R^2^ = 0.628, R^2^ = 0.682, R^2^ = 0.560, R^2^ = 0.643, R^2^ = 0.501, R^2^ = 0.545 respectively) with the MDA concentration of the culture media. An amino acid (Ala) and an acylcarnitine (tetradecadienoylcarnitine [C14:2]) had a negative correlation (R^2^ = − 0.565, R^2^ = − 0.603 respectively) with the MDA concentration (Fig. 8a).

An amino acid (Ala), an acylcarnitine (C3-DC) and two phosphatidylcholines (PC aa C42:6, PC ae C44:4) had a positive correlation (R^2^ = 0.763, R^2^ = 0.524, R^2^ = 0.534, R^2^ = 0.508 respectively) with the intracellular PC concentration. A biogenic amine (spermidine), an acylcarnitine (C4), a sphingomyelin (SM-OH C22:2) and a phosphatidylcholine (PC aa C32:3) were negatively correlated (R^2^ = − 0.857, R^2^ = − 0.610, R^2^ = − 0.530, R^2^ = − 0.501 respectively) with the PC concentration (Fig. 8b).

Only one metabolite, a lysophospatidylcholine (lysoPC a C24:0) was correlated positively (R^2^ = 0.502) with the IL-6 concentration (Fig. 8c). Also, only a phoshatidylcholine (PC ae C32:1) had a negative correlation (R^2^ = − 0.546) with the IL-8 content (Fig. 8d).

Three phosphatidylcholines (PC ae C38:5, PC aa C32:0, PC aa C42:1) had a positive correlation (R^2^ = − 0.622, R^2^ = − 0.571, R^2^ = − 0.521 respectively) with the GRP78 concentration (Fig. 8e).

Several metabolites showed positive correlation with the HSP27, including three amino acids (Orn, Asp, Ser) (R^2^ = 0.779, R^2^ = 0.731, R^2^ = 0.622 respectively), five biogenic amines (histamine, taurine, putrescine, spermidine, carnosine) (R^2^ = 0.752, R^2^ = 0.738, R^2^ = 0.607, R^2^ = 0.566, R^2^ = 0.561 respectively), an acylcarnitine (C3-DC) (R^2^ = 0.521) and four phosphatidylcholines (PC aa C36:1, PC aa C36:3, PC aa C40:6, PC ae C38:5) (R^2^ = 0.701, R^2^ = 0.578, R^2^ = 0.531, R^2^ = 0.522 respectively). Only two metabolites, an amino acid (asparagine [Asn]) and a phosphatidylcholine (PC ae C40:5) had a negative correlation (R^2^ = − 0.763, R^2^ = − 0.520 respectively) with the HSP27 level (Fig. 8f).

## Discussion

In the present study, chicken derived 3D hepatic cell cultures were used to demonstrate the effects of T-2 toxin to understand the molecular pathways which are affected by T-2 toxin. 3D cell cultures can provide a more proper model of the microenvironment and cellular function under in vivo conditions; in these cultures, cells keep their in vivo phenotype and characteristics as well as their ability to grow and interact with other cells and the ECM. In addition, 3D hepatic cell cultures can be maintained for longer than traditional 2D cell cultures^21,22^. In this study, we used hepatocyte—NP cell co-culture to mimic a near-physiological state. These models allow a better understanding of how cells respond to stress and inflammation, including cytokine production and the regulation of redox homeostasis^28^.

In order to investigate the adverse effects of T-2 toxin, 3D hepatocyte—NP cell co-cultures were treated with different concentrations of the toxin for 24 h. Metabolic activity of the cells was measured since T-2 toxin binds to various proteins and inhibits the function of several key enzymes involved in cellular catabolic processes, such as succinate dehydrogenase and mitochondrial NADH dehydrogenase, thereby causing cellular energy deficiency^6,18,29^. The metabolic activity of all treatment groups showed a steady yet significant decrease, which was inversely correlated with the increasing T-2 toxin concentration in the cell culture media. Our results indicate a mild decline (on average 19%) of hepatocellular metabolic activity triggered by T-2 toxin, which is in line with previous findings, such as the decreased viability of the liver cells in vitro both in the previous study of our research group on chicken 2D hepatocyte mono-cultures and hepatocyte—NP cell co-cultures as well as in porcine brain capillary endothelial cells (PBCEC) after T-2 toxin treatment described by Weidner et al.^6,30^.

To evaluate the toxin-associated necrosis of the cells, LDH activity was measured. LDH is rapidly released into the medium in case of membrane damage which could indicate necrosis. The LDH leakage of the hepatic cells was significantly increased by the 500 nM T-2 toxin treatment, suggesting impaired membrane integrity following the toxin exposure^28,31^. Measuring the metabolic activity of viable cells and the LDH activity of damaged cells can give a comprehensive picture about the cell viability and the toxicity of the toxin in both untreated and treated cell cultures.

Since T-2 toxin might induce oxidative stress, the amount of MDA, a lipid peroxidation product, and PC, a protein damage marker were measured in the cell culture media or in the cell lysates, respectively. Significant toxin-evoked decline was observed for both tested markers in certain groups. The highest T-2 toxin treatment effectively reduced the MDA level, whereas the PC concentration was significantly decreased after both the 500 nM and 1000 nM toxin exposure. These findings suggest that T-2 toxin induced protective mechanisms in the cells, resulting in the alleviation of oxidative stress as reflected by the assayed markers^6^. T-2 toxin has multiplex effects on the regulation of the stress response, it can either up- or downregulate the Nrf2 transcription factor in a dose- and time-dependent manner. If Nrf2 expression is elevated, it promotes the expression and production of antioxidant enzymes in response to oxidative stress, which could lead to the decrease of oxidative stress markers, such as MDA and PC^16,32^.

The immunomodulatory effects of T-2 toxin were investigated by measuring the IL-6 and IL-8 concentrations. The 100 nM T-2 toxin concentration significantly decreased the level of IL-6 in the culture media. The detected decrease in the IL-6 concentration triggered by low toxin concentration might be explained by the activation of autophagy. Autophagy has a major role in cytoprotection as it is a key regulator of the removal of irregular, non-functioning cellular components^33^. Previous studies indicate the activation of autophagy by T-2 toxin through autophagy-related gene 5 (ATG5) and mTOR regulation^34^. Furthermore, autophagy has been shown to prevent the release of inflammatory cytokines from macrophages during fibrogenesis in the liver, which may result in decreased levels of IL-6 in the cell culture media^35^. In addition, it is likely that IL-6 expression is regulated at the translational and post-translational level, given that IL-6 expression was unaffected at the mRNA level in a previous study in mice^36^.

The IL-8 release of the spheroids was significantly increased by the lowest toxin concentration. Previous studies have already shown that trichothecene mycotoxins, including T-2 toxin are able to activate the mitogen-activated protein kinase (MAPK) signaling pathway, through which they can stimulate the production of various pro-inflammatory cytokines that trigger functional disorders and apoptosis in order to protect the cells from harmful external stimuli^6,17,37^.

In order to investigate the effect of T-2 toxin and the potential oxidative stress on the ER stress, the levels of HSP27 and GRP78 were measured. In response to cytotoxic conditions, several changes happen in the ER leading to unfolded protein accumulation and aggregation. These changes are collectively called ER stress. Upon ER stress, certain signaling pathways are activated, such as the UPR and the ER overloaded response (EOR) pathways. Furthermore, some studies indicate that the ER cooperates with other organelles and plays a role in autophagy^38^. GRP78 is member of the HSP70 family, located in the membrane of the ER. It has a crucial role in the protein quality control of the ER and the regulation of the UPR^14,39^. The proteins responsible for the UPR are negatively regulated by the GRP78 in unstressed or healthy cells, but the elevated levels of unfolded proteins cause the dissociation of GRP78 from the URP transducer proteins, thereby releasing them from the inhibition^14^.

HSP27 is a molecular chaperone that belongs to the sHSPs and has a role in the early response upon oxidative and ER stress^13,15^. No significant changes were observed related to these parameters, indicating that the used T-2 toxin concentrations and incubation time did not induce ER stress in the cells. Hence, it can be suggested that ER stress is not primarily involved in the cellular effects of T-2 toxin on the avian liver.

Concerning the metabolome analysis, the abundance of 41 metabolites was significantly influenced by the toxin exposure. Among them, several significantly varied metabolites were phosphatidylcholines or their derivatives. This is consistent with the fact that T-2 toxin also affects lipid metabolism. On the one hand, due to its lipophilicity, T-2 toxin can damage the integrity and function of cell membranes, allowing it to rapidly reach various cellular components, leading to cytotoxicity^18,40^. Furthermore, T-2 toxin also stimulates ROS production, which induces the peroxidation of membrane lipids^41^. This may explain the decrease in the levels of certain phosphatidylcholines, as they are the main membrane-forming glycerophospholipid^42^. However, our results suggest that the rate of lipid peroxidation is reduced, probably due to the activation of the cytoprotective mechanisms described above in response to the highest toxin concentration.

One lysophosphatidylcholine—a phosphatidylcholine derivate—had a moderate positive correlation with the IL-6 content. Increased abundance of lysophosphatidylcholines has a lytic effect on the membrane and is often associated with inflammatory processes^42^. In addition, changes in their levels may also be indicative of processes caused by oxidative stress in cells as mentioned above^41,43^.

T-2 toxin also affects amino acid metabolism. In our experiments, the concentrations of four amino acids (proline, methionine, alanine, citrulline) and two biogenic amines (carnosine, spermidine) were significantly increased in the media by the treatments. A significant decrease in the levels of proline and methionine was observed in the control group compared to the cell-free medium. This suggests that the cells have taken up these two amino acids from their environment. For methionine, the increase in response to treatment did not surpass that measured in the cell-free medium, which may indicate that the uptake of the amino acid decreased after the treatment. However, in the case of proline, the increase in response to the highest toxin treatment exceeded the amount measured in the cell-free medium, suggesting an elevated secretion as a result of the treatment. Proline, alanine and citrulline play an important role in maintaining oxidative homeostasis. Several experiments have demonstrated that alanine was cytoprotective against oxidative stress^44^. It stimulated the expression of certain proteins involved in the antioxidant defense system in human endothelial cells and reduced LDH leakage in rat hepatocytes^45,46^. The hydroxyl radical scavenger property of citrulline and its DNA protective role during oxidative stress have also been described^47,48^. Proline can also effectively protect against oxidative stress in a variety of cell types^44^. Proline synthesis is enhanced during H_2_O_2_-induced oxidative stress and plays a role in the maintenance and protection of the intracellular glutathione redox system, possibly through its direct ROS scavenging property. In addition, elevated proline levels may activate glutathione synthesis, stabilize antioxidant enzymes or trigger signalling pathways that up-regulate certain cellular antioxidant processes^49^.

The levels of carnosine and spermidine were already increased in the control group compared to the cell-free medium, and this increase was further enhanced by the toxin treatments. Carnosine is a dipeptide composed of β-alanine and l-histidine^50^. It acts as a natural cellular antioxidant, neutralizing free radicals, cooperating with other antioxidants, regulating the function of antioxidant enzymes, and inhibiting lipid peroxidation and protein oxidation^51,52^. In addition, an in vivo study revealed that it was able to alleviate epithelial damage caused by another trichothecene mycotoxin, deoxynivalenol (DON), by reducing oxidative stress^50^.

Spermidine is an evolutionarily conserved polyamine with protective and lifespan-extending effects in mammals due to its anti-inflammatory and antioxidant properties. In addition, it improves mitochondrial metabolic function, proteostasis and chaperone activity^53,54^. In mice liver, spermidine improved the defense against oxidative stress both in vivo and in vitro^53^. Moreover, it reduced the production of pro-inflammatory cytokines through inhibition of nuclear translocation of nuclear factor κ-light-chain-enhancer of activated B cells (NFκB)^55^.

All these suggest that the elevated amino acid and biogenic amine levels are likely to be associated with oxidative stress caused by T-2 toxin, possibly mediating the activation of cellular protective mechanisms leading to maintained or even decreased MDA and PC levels.

Six amino acids (glutamine, glutamate, aspartate, lysine, serine, threonine) and two biogenic amines (putrescine, sarcosine) were significantly decreased in the media by the treatments. The levels of glutamine, glutamate and aspartate increased in the medium of control cells compared to the cell-free medium, indicating that the cells secrete these amino acids. The treatment-induced decrease did not exceed the amount of these amino acids in the cell-free medium, suggesting that the treatment caused a decrease in the release of these amino acids. In case of the glutamine, one possible reason for this decrease can be that glutamine is one of the precursors for the biosynthesis of proline in the mitochondria^56^. Accordingly, proline levels increased significantly during our experiment. Glutamine also plays a role in maintaining the oxidative homeostasis in the cells because of its ROS scavenging properties^57^. Glutamine concentration has also been shown to decrease during catabolic stress. This may be due to a decrease in the activity of the enzyme glutamine synthetase in response to ROS^58^. Glutamine may also serve as a precursor for the synthesis of glutathione^59^. This suggests that its abundance may be substantially reduced due to the activation of the glutathione system against the oxidative stress caused by T-2 toxin treatment.

Levels of glutamate, an intermediate of the glutamine metabolism, and aspartate were also reduced by the toxin treatment. Both glutamate and aspartate are key substrates for other biologically active molecules such as glutamine, glutathione, proline, ornithine and arginine, which may also explain why their release into the medium was reduced in the samples^60–62^.

Interestingly, the level of another amino acid, lysine, was also significantly decreased by the T-2 toxin treatments, but the amount of α-aminoadipic acid, the end product of the lysine oxidation, was significantly increased^63^. α-aminoadipic acid is an appropriate indicator of in vivo protein oxidation during oxidative stress, which has long been recognized and used in the study of various human diseases and aging^64^. Therefore, the increase in α-aminoadipic acid level may confirm the development of oxidative stress in the hepatic cells.

Serine concentration was also decreased by the T-2 toxin treatment. Serine plays an important role in glycolysis as well as purine synthesis, the one-carbon metabolic cycle and glutathione synthesis. It is a non-essential amino acid, as it can be produced in sufficient quantities by the organism under physiological conditions. However, several studies have described that a serine deficiency can occur under oxidative stress^65^. The consequence of this is a stronger response to oxidative stress and higher ROS concentrations^65,66^. During oxidative stress, the one-carbon cycle switches from methylation to transsulfuration in order to synthesize more glutathione, where serine may also serve as a precursor^65^.

In addition to a decrease in serine, a decrease in threonine content has also been described under oxidative stress^66,67^. Threonine is an essential amino acid for chickens^67^. Adequate levels of threonine are important for proper growth and threonine also plays an important role as a precursor in the synthesis of several amino acids^67^. Furthermore, serine/threonine kinases are often associated with the response during oxidative stress. These serine/threonine kinases can phosphorylate either serine or threonine and play a key role in the induction of cell growth, proliferation and apoptosis^68^. Such serine/threonine kinase is the Akt, which phosphorylates mTOR^69^. This pathway can be inhibited by several mycotoxins, including T-2 toxin, thereby regulating autophagy^68,69^.

The amount of putrescine and sarcosine increased in the medium of control cells compared to the cell-free medium, indicating their release by the cells. This secretion was reduced by T-2 toxin treatment. Putrescine (1,4-diaminobutane) is a naturally occurring polyamine, along with spermidine and spermine^70^. Putrescine plays an important role in proliferation and cell growth as well as in the regulation of transcription and translation^71,72^. Furthermore, it serves as a precursor for the synthesis of other polyamines such as spermidine^72^. Accordingly, the level of putrescine in media samples decreased, while the concentration of spermidine increased significantly, which may indicate a putrescine-spermidine conversion.

The concentration of sarcosine was significantly decreased by the toxin treatment. The methylation of sarcosine (*N*-methylglycine) leads to the formation of trimethylglycine, a metabolite that typically accumulates during oxidative stress and has a protective role against it^73^. Moreover, sarcosine typically forms complexes with metals, which can also prevent nucleic acid oxidation^73,74^. Both of these processes could lead to a decline in the sarcosine content.

It was found that the concentration of eight acylcarnitines decreased, while three increased significantly after the T-2 toxin treatment. Different acylcarnitines also showed both moderate positive correlations with PC and HSP27, while another had a moderate negative correlation with MDA. L-carnitine is found in free form or as acylcarnitine, an esterified form in the cytoplasm of cells^75^. l-Carnitine and acylcarnitines play a key role in lipid metabolism, as the carnitine shuttle system transports fatty acids into the mitochondria where β-oxidation occurs. In this process, acylcarnitine crosses the mitochondrial membrane, where the fatty acids are detached, and the free carnitine is returned to the cytoplasm^76^. l-Carnitine also has a role in stimulating the production of antioxidant enzymes and reducing oxidative stress and inflammatory cytokine release^77^. In addition, acylcarnitines are known to have antioxidant activity, but the underlying mechanism is not yet fully understood^78^.

Histamine and taurine had a strong positive correlation with the HSP27 levels. Histamine is a key mediator of inflammation and has an important role in a number of other cellular processes, including the regulation of the stress response^79^. Previous studies have already described its role in the phosphorylation and activation of HSP27, which may support why their levels were so closely correlated in the examined samples after the toxin treatment^79,80^.

Taurine is a biogenic amine with a long-established cytoprotective effect. It has been shown to play a role in ER stress defence through downregulation of UPR-related proteins such as GRP78^81,82^. Moreover, in chickens exposed to taurine, it was observed that the expression of certain heat shock proteins was reduced in vivo^83^. In addition, it can reduce inflammation by decreasing the levels of several pro-inflammatory cytokines, and has an antioxidant effect by stimulating the expression of the transcription factor Nrf2 as well as reducing lipid peroxidation^81,84^.

Asparagine concentration was negatively correlated with that of HSP27. This could be explained by the fact that transcription of the asparagine synthetase enzyme is enhanced by ER stress via the UPR^85,86^. Asparagine synthetase catalyses the synthesis of asparagine and glutamate, and therefore enhances asparagine formation in the presence of ER stress^85^.

Ornithine level was positively correlated with the concentration of MDA and HSP27. Ornithine decarboxylase is an important enzyme in polyamine biosynthesis, converting ornithine to putrescine, which, as mentioned above, then serves as a precursor for the synthesis of spermidine^72,87^. The function of the enzymes catalysing polyamine synthesis is usually affected by various stress situations, such as exposure to T-2 toxin^87,88^. Among them, the transcription of the ornithine decarboxylase gene was significantly upregulated by chemically induced oxidative stress in vitro^87^.

Symmetric dimethylarginine (SDMA) and asymmetric dimethylarginine (ADMA) are methylated derivatives of l-Arginine^89^. Both are toxic, non-proteinogenic amino acids capable of inhibiting nitric oxide (NO) production^90,91^. In addition, SDMA has a possible proinflammatory effect and may also induce ROS formation^90^. This may be related to our finding that the amount of SDMA was positively correlated with the concentration of an oxidative stress marker, MDA.

## Conclusion

T-2 toxin decreased the metabolic activity of cells and increased the extent of cell membrane damage, therefore had a negative effect on the viability of liver cells. Our results suggest that T-2 toxin may also alter the cellular oxidative homeostasis through the promotion of ROS production as well as the regulation of the Nrf2 transcription factor and may influence immune function including the release of pro-inflammatory cytokines such as IL-6 and IL-8. According to our results, lipid and amino acid metabolism were also affected by the toxin leading to remarkable alterations in the metabolome of cell cultures. In conclusion, several cellular processes, such as the inflammatory and oxidative stress response or the metabolic profile of hepatic spheroids were modulated by T-2 toxin exposure, and investigating the complex interactions of these processes is a good basis for future studies on the mechanism of action of T-2 toxin. Furthermore, our applied cell culture model can serve as a promising tool for the investigation of various further mycotoxins as well as other toxic agents in the future.

## Materials and methods

All reagents used in the present study were purchased from Merck KGaA (Darmstadt, Germany) except when otherwise specified. Animal procedures were performed according to the international and national law as well as the institutional guidelines and were confirmed by the Government Office of Zala County, Food Chain Safety, Plant Protection and Soil Conservation Directorate, Budapest, Hungary (permission number: ZAI/040/00522-7/2020). The study was conducted following the ARRIVE guidelines 2.0 (https://arriveguidelines.org/). The animals were fed and reared in accordance with the requirements of the Ross Technology^92^.

### Cell isolation and culturing conditions

The cells were isolated from a 3-week-old Ross 308 male broiler chicken (obtained from Gallus Poultry Farming and Hatching Ltd, Devecser, Hungary) as described by Mackei et al.^28^. Under CO_2_ narcosis, the animal was decapitated, and the liver was perfused through the gastropancreaticoduodenal vein by three-step perfusion. During the procedure, all buffers were heated to 40 °C and freshly oxygenated with Carbogen (95% O_2_, 5% CO_2_), the velocity of the perfusion was set to 30 ml/min.

In the first step, the liver was washed with 150 ml of 0.5 nM ethylene glycol tetraacetic acid (EGTA) containing Hanks' Balanced Salt Solution (HBSS), followed by 150 ml of EGTA-free HBSS. As a final step, 130 ml of MgCl_2_ and CaCl_2_ (both 7 mM) containing HBSS supplemented with 1 mg/ml type IV collagenase (Nordmark, Uetersen, Germany) was rinsed to ensure that the extracellular matrix was degraded and the organ was disrupted.

After excision of the liver, the Glisson's capsule was opened and the freshly gained cells were suspended in 50 ml of ice-cold bovine serum albumin (BSA, 2.5%) containing HBSS. The suspension was filtered through 3 layers of sterile gauze to remove any cell aggregate leftovers and undigested interstitial tissue. The resulting cell suspension was incubated on ice for 50 min.

Afterward, the fractions containing hepatocytes and NP cells were separated by multistep differential centrifugation. The suspension was centrifuged three times for 3 min at 100×*g*. Between the steps, the NP cell containing supernatant was collected separately and the hepatocyte enriched sediment was resuspended in Williams' Medium E supplemented with 0.22% NaHCO_3_, 50 mg/ml gentamycin, 2 mM glutamine, 4 µg/l dexamethasone, 20 IU/l insulin and 5% foetal bovine serum (FBS). At the end of centrifugation, a purified hepatocyte fraction free of NP cells was obtained.

To separate the NP cell containing fraction, the previously collected supernatants were centrifuged first at 350×*g* for 10 min in order to remove residual hepatocytes and red blood cells, and then the supernatant was centrifuged at 800×*g* for 10 min, gaining the final sediment containing the NP cells.

Cell viability and total cell number were determined in Bürker chambers by trypan blue exclusion test. The number of viable cells exceeded 90% for both cell types. The appropriate cell concentrations were adjusted to 5 × 10^5^ cells/ml and the hepatocyte:NP cell ratio was set to 6:1.

All the needed equipment and chemicals for the preparation of magnetic 3D cell cultures were purchased from Greiner Bio-One Hungary Ltd (Mosonmagyaróvár, Hungary). In order to magnetize the cells, 800 µl of magnetic nanoparticles (NanoShuttle™-PL) were added to 8 ml of hepatocyte—NP cell co-culture suspension. These nanoparticles are biocompatible and have no effect on the viability and proliferation of the cells^25–27^. The cells were then seeded onto a 96-well cell repellent plate and were incubated at 37 °C for 1 h. During this time, the magnetic nanoparticles bound to the cell membrane. Afterwards, the plate was placed on top of a magnetic drive with small magnets under each well of the plate (Spheroid Drive) and was incubated for 48 h at 37 °C in 100% relative humidity with 5% CO_2_. The culture medium was changed to serum-free medium after 24 h with the use of a Holding Drive.

### Treatment of the cell cultures

After 48 h incubation, the 3D cell cultures were exposed to culture media supplemented with 0 (control), 100, 500 or 1000 nM T-2 toxin for 24 h. Samples were collected at the end of the incubation from the cell culture media, and the cells were lysed by intermittent sonication (1/s) in 40 µl of M-PER buffer for 5 s using a Bandelin Sonopuls HD 2200 homogenizer (Bandelin Electronic GmbH & Co. KG, Berlin, Germany). The samples were stored at − 80 °C until further analysis. The control, untreated spheroids have been fixed in 10% buffered formalin solution and after embedding and sectioning the slides they were stained with haematoxylin and eosin to examine the spheroid morphology.

### Measurements

#### Metabolic activity of the cells

The metabolic activity of the cells was evaluated by CCK-8 assay (Dojindo Molecular Technologies, Rockville, MD, USA) according to the manufacturer’s protocol, detecting the amount of NADH + H^+^ produced in the catabolic processes of the cells. 10 µl CCK-8 reagent and 100 µl of the cell culture media were added to each well of a 96-well plate, and after 4 h of incubation, the absorbance was measured at 450 nm with a Multiskan GO 3.2 reader (Thermo Fisher Scientific, Waltham, MA, USA).

#### Lactate dehydrogenase activity of the cells

To investigate the damage of the cell membranes, the extracellular activity of LDH was determined by LDH Activity Assay Kit. Measurements were performed following the manufacturer's instructions on 96-well plates. First, 50 µl of Master Reaction Mix (48 µl LDH Assay Buffer, 2 µl LDH Substrate Mix) was added to 50 µl of sample and absorbances were measured at 450 nm using a Multiskan GO 3.2 reader (Thermo Fisher Scientific, Waltham, MA, USA). To determine the LDH activity, absorbances were read at 5 min intervals until the most active sample exceeded the highest standard concentration. LDH activity was then calculated according to the manufacturer's protocol.

#### Cellular inflammation and oxidative stress

All measurements concerning stress and inflammatory markers were performed using chicken specific ELISA kits (MyBioSource, San Diego, CA, USA) according to the manufacturer’s instructions. To investigate the oxidative stress, the concentration of a lipid peroxidation marker, MDA was measured from the cell culture media. The protein damage caused by oxidative stress was detected by the determination of PC content from the cell lysates. In order to determine the effects of T-2 toxin on cellular inflammation, the concentrations of two pro-inflammatory cytokines, IL-6 and IL-8 were assayed from cell-free supernatants. ER stress was evaluated by measuring the concentration of GRP78 and HSP27 from the media.

#### Metabolome analysis

Metabolome analysis of the media samples was carried out using the AbsoluteIDQ p180 Kit (Biocrates Life Sciences AG, Innsbruck, Austria) in accordance with the manufacturer's instructions. This kit detects and quantifies up to 188 metabolites belonging to 5 separate compound classes: acylcarnitines (40), proteinogenic and modified amino acids (19), glycerophospho- and sphingolipids (76 phosphatidylcholines, 14 lysophosphatidylcholines, 15 sphingomyelins), biogenic amines (19) and hexoses (1) (Supplementary Table 3). The amino acids and biogenic amines were analyzed by liquid chromatography–mass spectrometry (LC–MS/MS) and the acylcarnitines, phosphatidylcholines (including lysophosphatidylcholines), sphingomyelines and hexoses were assesed by flow injection analysis–mass spectrometry (FIA–MS/MS) at the Core Facility of the University of Hohenheim (Stuttgart, Germany)^93^. 10 µl of culture medium with internal standard, PBS and calibration standards in a multititer plate and dried under nitrogen (nitrogen evaporator 96 well plate, VLM GmbH, Bielefeld, Germany) for 30 min. Afterwards, the metabolites were derivatized with 5% phenylisothiocyanate (PITC) for 25 min at room temperature and dried under nitrogen flow for 60 min. For extraction, first 300 µl of extraction solvent (5 mM ammonium acetate in methanol) was added and then incubated with shaking at 450 rpm (Thermomixer comfort Eppendorf, Hamburg, Germany) for 30 min at room temperature. The extraction solvent was then eluted using a nitrogen pressure unit. Thereafter, 50 µl of filtrate was removed and transferred to a fresh multititer deepwell plate where it was diluted with 450 µl of 40% HPLC grade methanol for LC–MS analysis. For FIA-MS/MS analysis, 10 µl of filtrate and 490 µl of mobile phase solvent were added to a new 96-well microplate. Both measurements were performed with a QTRAP mass spectrometer applying electrospray ionization (ESI) (AB Sciex API 5500Q-TRAP). MS was coupled to an ultra-performance liquid chromatography (UPLC) (Agilent 1290, Agilent Technologies Deutschland GmbH, Waldbronn, Germany). For LC–MS, the metabolites were separated by a hyphenated reverse phase column (Waters, ACQUITY BEH C18, 2.1 × 75 mm, 1.7 µm; Waters, Milford, United States) preceded with a precolumn (Security Guard, Phenomenex, C18, 4 × 3 mm; Phenomenex, Aschaffenburg, Germany) applying a gradient of solvent A (formic acid 0. 2% in water) and solvent B (formic acid 0.2% in acetonitrile) over 7.3 min (0.45 min 0% B, 3.3 min 15% B, 5.9 min 70% B, 0.15 min 70% B, 0.5 min 0% B) at a flow rate of 800 µl/min. Oven temperature was 50 °C. For LC–MS analysis 5 µl, for FIA analysis 2 × 20 µl were subjected for measurements in both positive and negative mode. Identification and quantification were achieved by multiple reaction monitoring (MRM), which was standardized by applying spiked-in isotopically labelled standards in positive and negative mode, respectively. A calibrator mix consisting of seven different concentrations was used for calibration. Quality controls were derived from lyophilized human plasma samples at 3 different concentrations. For FIA, an isocratic method was used (100/% organic running solvent) with varying flow conditions (0 min, 30 µl/min; 1.6 min, 30 µl/min; 2.4 min, 200 µl/min; 2.8 min, 200 µl/min; 3 min, 30 µl/min) and the MS settings were as follows: scan time 0.5 s, IS voltage for positive mode 5500 V, for negative mode − 4500 V, source temperature 200 °C, nitrogen as collision gas medium. During LC–MS the corresponding parameters were: scan time 0.5 s, source temperature 500 °C, nitrogen as collision gas medium.

All reagents used for processing and analysis were LC–MS grade unless otherwise specified. Milli-Q Water ultrapure was used fresh after preparation with a high-purity water system (Merck KGaA, Darmstadt, Germany). LC–MS grade acetonitrile, methanol, pyridine and formic acid were purchased from Merck KGaA, and PITC as well as ammonium acetate from Sigma Aldrich Chemie GmbH (Steinheim, Germany).

Raw data obtained with Analyst software (AB Sciex, Framingham, MA, USA) were processed with MetIDQ software, an integrated member of the AbsoluteIDQ p180 Kit. This streamlines data analysis by automated calculation of metabolite concentrations providing quality measures and quantification. For fully quantitative measurements, the lower limit of quantification (LLOQ) was determined in plasma experimentally by Biocrates Life Sciences AG.

### Statistical analysis

The statistical analysis was performed using the R Statistical Software (v4.1.1; R Core Team 2021). Each treatment group contained 15 replicates (n = 15/group). During the analysis, treatment groups were compared to the control group. Shapiro–Wilk test and Levene’s test were used to verify normal distribution and homogeneity of variance, respectively. Differences between various groups were assessed using one-way analysis of variance (ANOVA) and Dunett’s post hoc tests for pairwise comparisons. Results were expressed as mean ± standard error of the mean (SEM). Differences were considered significant at* p* < 0.05. Results were visualized using Graphad Prism version 9.1.2 for Windows (GraphPad Software, San Diego, CA, USA). Results were visualised with relative values and Supplementary Table 1 shows the corresponding group means.

The data from the metabolome analysis were processed with MetaboAnalyst 5.0 after log transformation and Pareto scaling normalization. Metabolites with significantly altered abundance in different treatment groups (toxin-exposed cells compared to the control group) were determined using the ANOVA function with Tukey’s HSD (honestly significant difference) for pairwise comparisons. Further, the metabolome of the control group was also compared to that of cell-free culture media to monitor the spheroid-associated metabolic changes independently from the toxin exposure. In order to determine the significantly changed metabolites between the cell-free medium and control group, unpaired t-test was used. The correlation between the parameters measured by chicken specific ELISA tests (MDA, PC, IL-6, IL-8, GRP78, HSP27) and metabolites derived from the metabolomics approach was also investigated using the Pattern Hunter function of the program. The correlation coefficients (R^2^) were interpreted according to a guide for medical research^94^.

### Supplementary Information

Supplementary Legends.

Supplementary Table 1.

Supplementary Table 2.

Supplementary Table 3.

## Data Availability

All data generated or analyzed during this study are included in this published article (and its Supplementary Information files).

## References

[1] Li Y (2011). T-2 toxin, a trichothecene mycotoxin: Review of toxicity, metabolism, and analytical methods. J. Agric. Food Chem..

[2] Galbenu-Morvay PL, Trif A, Damiescu L, Simion G (2011). T-2 toxin occurrence in cereals and cereal-based foods. Bull. Univ. Agric. Sci. Vet. Med. Cluj-Napoca. Agric..

[3] Swanson SP (1987). Metabolism of three trichothecene mycotoxins, T-2 toxin, diacetoxyscirpenol and deoxynivalenol, by bovine rumen microorganisms. J. Chromatogr..

[4] Kuca K, Dohnal V, Jezkova A, Jun D (2008). Metabolic pathways of T-2 toxin. Curr. Drug Metab..

[5] Kiš M (2021). A two-year occurrence of fusarium T-2 and HT-2 toxin in croatian cereals relative of the regional weather. Toxins.

[6] Mackei M (2020). Cellular effects of T-2 toxin on primary hepatic cell culture models of chickens. Toxins (Basel).

[7] Awad WA (2008). The impact of the fusarium toxin deoxynivalenol (DON) on poultry. Int. J. Poultry Sci..

[8] Awad WA (2012). Genotoxic effects of deoxynivalenol in broiler chickens fed low-protein feeds. Poultry Sci..

[9] Osselaere A (2013). Toxic effects of dietary exposure to T-2 toxin on intestinal and hepatic biotransformation enzymes and drug transporter systems in broiler chickens. Food Chem. Toxicol..

[10] Dai C (2019). T-2 toxin neurotoxicity: Role of oxidative stress and mitochondrial dysfunction. Arch Toxicol..

[11] Liu A (2019). DNA methylation is involved in pro-inflammatory cytokines expression in T-2 toxin-induced liver injury. Food Chem. Toxicol..

[12] Yao L (2015). Roles of oxidative stress and endoplasmic reticulum stress in selenium deficiency-induced apoptosis in chicken liver. Biometals.

[13] Burban A, Sharanek A, Guguen-Guillouzo C, Guillouzo A (2018). Endoplasmic reticulum stress precedes oxidative stress in antibiotic-induced cholestasis and cytotoxicity in human hepatocytes. Free Radic. Biol. Med..

[14] Chaudhari N, Talwar P, Parimisetty A, Lefebvre d’Hellencourt C, Ravanan P (2014). A molecular web: Endoplasmic reticulum stress, inflammation, and oxidative stress. Front. Cell. Neurosci.

[15] Ito H (2005). Endoplasmic reticulum stress induces the phosphorylation of small heat shock protein, Hsp27. J. Cell. Biochem..

[16] Deyu H (2018). Protective mechanisms involving enhanced mitochondrial functions and mitophagy against T-2 toxin-induced toxicities in GH3 cells. Toxicol. Lett..

[17] Pomothy JM (2020). Beneficial effects of rosmarinic acid on IPEC-J2 cells exposed to the combination of deoxynivalenol and T-2 toxin. Mediators Inflamm..

[18] Wan Q, Wu G, He Q, Tang H, Wang Y (2015). The toxicity of acute exposure to T-2 toxin evaluated by the metabonomics technique. Mol. BioSyst..

[19] Kenéz Á, Dänicke S, Rolle-Kampczyk U, von Bergen M, Huber K (2016). A metabolomics approach to characterize phenotypes of metabolic transition from late pregnancy to early lactation in dairy cows. Metabolomics.

[20] Desai PK, Tseng H, Souza GR (2017). Assembly of hepatocyte spheroids using magnetic 3D cell culture for CYP450 inhibition/induction. Int. J. Mol. Sci..

[21] van Zijl F, Mikulits W (2010). Hepatospheres: Three dimensional cell cultures resemble physiological conditions of the liver. World J. Hepatol..

[22] Souza AG (2018). Comparative assay of 2D and 3D cell culture models: Proliferation, gene expression and anticancer drug response. Curr. Pharm. Des..

[23] Tseng, H. & Souza, G. R. M3D cell culture: Bicompatibility of nanoshuttle-PL and the magnetic field. https://www.gbo.com/fileadmin/media/GBO-International/01_Downloads_BioScience/SALES_White_Papers/F075068_m3D_White_Paper_Biocompatibility_Nanoshuttle_EN.pdf. (2022).

[24] Vékony V, Matta C, Pál P, Szabó IA (2022). Structural and magnetic characterisation of a biocompatible magnetic nanoparticle assembly. J. Magnet. Magnet. Mater..

[25] Daquinag AC, Souza GR, Kolonin MG (2013). Adipose tissue engineering in three-dimensional levitation tissue culture system based on magnetic nanoparticles. Tissue Eng. Part C Methods.

[26] Tseng H (2013). Assembly of a three-dimensional multitype bronchiole coculture model using magnetic levitation. Tissue Eng. Part C Methods.

[27] Tseng H (2014). A three-dimensional co-culture model of the aortic valve using magnetic levitation. Acta Biomater..

[28] Mackei M (2020). Effects of acute heat stress on a newly established chicken hepatocyte-nonparenchymal cell co-culture model. Animals (Basel).

[29] Adhikari M (2017). T-2 mycotoxin: Toxicological effects and decontamination strategies. Oncotarget.

[30] Weidner M (2013). Influence of T-2 and HT-2 toxin on the blood-brain barrier in vitro: New experimental hints for neurotoxic effects. PLoS One.

[31] Yang L (2016). Toxicity and oxidative stress induced by T-2 toxin and HT-2 toxin in broilers and broiler hepatocytes. Food Chem. Toxicol..

[32] Kozieł MJ, Kowalska K, Piastowska-Ciesielska AW (2021). Nrf2: A main responsive element in cells to mycotoxin-induced toxicity. Arch Toxicol..

[33] Sebők C (2021). Two sides to every question: Attempts to activate chicken innate immunity in 2D and 3D hepatic cell cultures. Cells.

[34] Guo P (2018). Brain damage and neurological symptoms induced by T-2 toxin in rat brain. Toxicol. Lett..

[35] Weiskirchen R, Tacke F (2019). Relevance of autophagy in parenchymal and non-parenchymal liver cells for health and disease. Cells.

[36] Dugyala RR, Sharma RP (1997). Alteration of major cytokines produced by mitogen-activated peritoneal macrophages and splenocytes in T-2 toxin-treated male CD-1 mice. Environ. Toxicol. Pharmacol..

[37] Pestka JJ (2010). Deoxynivalenol-induced proinflammatory gene expression: Mechanisms and pathological sequelae. Toxins.

[38] Faitova J, Krekac D, Hrstka R, Vojtesek B (2006). Endoplasmic reticulum stress and apoptosis. Cell. Mol. Biol. Lett.

[39] Yi Y (2018). Endoplasmic reticulum stress is involved in the T-2 toxin-induced apoptosis in goat endometrium epithelial cells: ER STRESS AND T-2-INDUCED APOPTOSIS. J. Appl. Toxicol..

[40] Wan Q (2016). Systemic metabolic responses of broiler chickens and piglets to acute T-2 toxin intravenous exposure. J. Agric. Food Chem..

[41] Ossani G (2007). Oxidative damage lipid peroxidation in the kidney of choline-deficient rats. Front. Biosci..

[42] Taylor LA, Arends J, Hodina AK, Unger C, Massing U (2007). Plasma lyso-phosphatidylcholine concentration is decreased in cancer patients with weight loss and activated inflammatory status. Lipids Health Dis..

[43] Frey B (2000). Increase in fragmented phosphatidylcholine in blood plasma by oxidative stress. J. Lipid Res..

[44] Li H-T (2013). Oxidative stress parameters and anti-apoptotic response to hydroxyl radicals in fish erythrocytes: Protective effects of glutamine, alanine, citrulline and proline. Aquat. Toxicol..

[45] Grosser N (2004). Antioxidant action of L-alanine: Heme oxygenase-1 and ferritin as possible mediators. Biochem. Biophys. Res. Commun..

[46] Ishizaki-Koizumi S, Sonaka I, Fujitani S, Nishiguchi S (2002). Mechanisms of the protective effect of l-alanine to d-galactosamine-induced hepatocellular injury: Comparative studies of l-alanine and pyruvate. Biochem. Biophys. Res. Commun..

[47] Akashi K, Miyake C, Yokota A (2001). Citrulline, a novel compatible solute in drought-tolerant wild watermelon leaves, is an efficient hydroxyl radical scavenger. FEBS Lett..

[48] Kaore SN, Amane HS, Kaore NM (2013). Citrulline: Pharmacological perspectives and its role as an emerging biomarker in future. Fundamental Clin. Pharmacol..

[49] Krishnan N, Dickman MB, Becker DF (2008). Proline modulates the intracellular redox environment and protects mammalian cells against oxidative stress. Free Radic. Biol. Med..

[50] Zhou J (2021). l-Carnosine protects against deoxynivalenol-induced oxidative stress in intestinal stem cells by regulating the Keap1/Nrf2 signaling pathway. Mol. Nutr. Food Res..

[51] Kalaz EB (2014). Carnosine and taurine treatments decreased oxidative stress and tissue damage induced by d-galactose in rat liver. J. Physiol. Biochem..

[52] Aydın AF, Küçükgergin C, Özdemirler-Erata G, Koçak-Toker N, Uysal M (2010). The effect of carnosine treatment on prooxidant–antioxidant balance in liver, heart and brain tissues of male aged rats. Biogerontology.

[53] Campreciós G (2021). Spermidine supplementation protects the liver endothelium from liver damage in mice. Nutrients.

[54] Yue F (2017). Spermidine prolongs lifespan and prevents liver fibrosis and hepatocellular carcinoma by activating MAP1S-mediated autophagy. Cancer Res..

[55] Adhikari R (2021). Spermidine prevents ethanol and lipopolysaccharide-induced hepatic injury in mice. Molecules.

[56] Burke L (2020). The Janus-like role of proline metabolism in cancer. Cell Death Discov..

[57] Wu L (2013). Effects of dietary arginine and glutamine on alleviating the impairment induced by deoxynivalenol stress and immune relevant cytokines in growing pigs. PLOS ONE.

[58] Matés JM, Pérez-Gómez C, de Castro IN, Asenjo M, Márquez J (2002). Glutamine and its relationship with intracellular redox status, oxidative stress and cell proliferation/death. Int. J. Biochem. Cell Biol..

[59] Curi R (2005). Molecular mechanisms of glutamine action. J. Cell Physiol..

[60] Boutry C (2012). Decreased glutamate, glutamine and citrulline concentrations in plasma and muscle in endotoxemia cannot be reversed by glutamate or glutamine supplementation: A primary intestinal defect?. Amino Acids.

[61] Riby JE, Hurwitz RE, Kretchmer N (1990). Development of ornithine metabolism in the mouse intestine. Pediatr. Res..

[62] Duan J (2016). Dietary supplementation with l-glutamate and l-aspartate alleviates oxidative stress in weaned piglets challenged with hydrogen peroxide. Amino Acids.

[63] Díaz-Velasco S, Delgado J, Peña FJ, Estévez M (2022). Protein oxidation marker, α-amino adipic acid, impairs proteome of differentiated human enterocytes: Underlying toxicological mechanisms. Biochimica et Biophysica Acta BBA Proteins Proteom..

[64] Estaras M, Ameur FZ, Estévez M, Díaz-Velasco S, Gonzalez A (2020). The lysine derivative aminoadipic acid, a biomarker of protein oxidation and diabetes-risk, induces production of reactive oxygen species and impairs trypsin secretion in mouse pancreatic acinar cells. Food Chem. Toxicol..

[65] Zhou X (2017). Serine alleviates oxidative stress via supporting glutathione synthesis and methionine cycle in mice. Mol. Nutr. Food Res..

[66] Yin J (2015). Effects of dietary supplementation with glutamate and aspartate on diquat-induced oxidative stress in piglets. PLoS One.

[67] Shirisha DR, Bu DU, Prashanth DK (2018). Effect of L-threonine supplementation on broiler chicken: A review. Pharma Innovation.

[68] Wang J, Yang C, Yuan Z, Yi J, Wu J (2018). T-2 Toxin exposure induces apoptosis in TM3 cells by inhibiting mammalian target of rapamycin/serine/threonine protein Kinase(mTORC2/AKT) to promote Ca^2+^ production. Int. J. Mol. Sci..

[69] Yin H (2020). T-2 toxin induces oxidative stress, apoptosis and cytoprotective autophagy in chicken hepatocytes. Toxins.

[70] Raina A, Jänne J (1975). Physiology of the natural polyamines putrescine, spermidine and spermine. Med. Biol..

[71] Dasdelen D, Cetin N, Menevse E, Baltaci AK, Mogulkoc R (2023). Effects of putrescine on oxidative stress, spermidine/spermine-N(1)-acetyltransferase, inflammation and energy levels in liver and serum in rats with brain ischemia-reperfusion. Physiol. Int..

[72] Qian Z-G, Xia X-X, Lee SY (2009). Metabolic engineering of *Escherichia coli* for the production of putrescine: A four carbon diamine. Biotechnol. Bioeng..

[73] Zemanová V, Pavlík M, Pavlíková D (2017). Cadmium toxicity induced contrasting patterns of concentrations of free sarcosine, specific amino acids and selected microelements in two Noccaea species. PLOS ONE.

[74] Tanas A (2022). In vitro and in vivo neuroprotective effects of sarcosine. BioMed. Res. Int..

[75] Ishikawa H (2014). L-Carnitine prevents progression of non-alcoholic steatohepatitis in a mouse model with upregulation of mitochondrial pathway. PLoS ONE.

[76] Indiveri C (2011). The mitochondrial carnitine/acylcarnitine carrier: Function, structure and physiopathology. Mol. Aspects Med..

[77] Li S, Gao D, Jiang Y (2019). Function, detection and alteration of acylcarnitine metabolism in hepatocellular carcinoma. Metabolites.

[78] Jones LL, McDonald DA, Borum PR (2010). Acylcarnitines: Role in brain. Progress Lipid Res..

[79] Delitheos B, Papamichael K, Tiligada E (2010). Histamine modulates the cellular stress response in yeast. Amino Acids.

[80] Rada CC (2021). Heat shock protein 27 activity is linked to endothelial barrier recovery after proinflammatory GPCR-induced disruption. Sci. Signal..

[81] Baliou S (2021). Protective role of taurine against oxidative stress (Review). Mol. Med. Rep..

[82] Pan C, Giraldo GS, Prentice H, Wu J-Y (2010). Taurine protection of PC12 cells against endoplasmic reticulum stress induced by oxidative stress. J. Biomed. Sci..

[83] Belal SA, Kang DR, Cho ESR, Park GH, Shim KS (2018). Taurine reduces heat stress by regulating the expression of heat shock proteins in broilers exposed to chronic heat. Braz. J. Poult. Sci..

[84] Surai PF, Kochish II, Kidd MT (2020). Taurine in poultry nutrition. Animal Feed Sci. Technol..

[85] Lomelino CL, Andring JT, McKenna R, Kilberg MS (2017). Asparagine synthetase: Function, structure, and role in disease. J. Biol. Chem..

[86] Pan Y, Chen H, Siu F, Kilberg MS (2003). Amino acid deprivation and endoplasmic reticulum stress induce expression of multiple activating transcription factor-3 mRNA species that, when overexpressed in HepG2 cells, modulate transcription by the human asparagine synthetase promoter *. J. Biol. Chem..

[87] Smirnova OA (2012). Chemically induced oxidative stress increases polyamine levels by activating the transcription of ornithine decarboxylase and spermidine/spermine-N1-acetyltransferase in human hepatoma HUH7 cells. Biochimie.

[88] Meloche JL, Smith TK (1995). Altered tissue amino acid metabolism in acute T-2 toxicosis. Proc. Soc. Exp. Biol. Med..

[89] Arlouskaya Y (2019). Asymmetric dimethylarginine (ADMA) and symmetric dimethylarginine (SDMA) concentrations in patients with obesity and the risk of obstructive sleep apnea (OSA). J. Clin. Med..

[90] Tain Y-L, Hsu C-N (2017). Toxic dimethylarginines: Asymmetric dimethylarginine (ADMA) and symmetric dimethylarginine (SDMA). Toxins.

[91] Nijveldt RJ (2004). Gut and liver handling of asymmetric and symmetric dimethylarginine in the rat under basal conditions and during endotoxemia. Liver Int..

[92] Aviagen. Ross Broiler Management Handbook. https://aviagen.com/assets/Tech_Center/Ross_Broiler/Ross-BroilerHandbook2018-EN.pdf. (2018).

[93] Bäßler SC (2021). Association between alterations in plasma metabolome profiles and laminitis in intensively finished Holstein bulls in a randomized controlled study. Sci. Rep..

[94] Mukaka M (2012). A guide to appropriate use of Correlation coefficient in medical research. Malawi Med. J..

