# Supplementary material for: Investigation of the effects of T-2 toxin in chicken-derived three-dimensional hepatic cell cultures
Source: Sci Rep. 2024 Jan 12;14:1195. doi: 10.1038/s41598-024-51689-1 (PMC10786837; doi:10.1038/s41598-024-51689-1)
Supplement: Supplementary file 1 — Supplementary Legends. [file 41598_2024_51689_MOESM1_ESM.docx]

**Supplementary Table 1.** Means of the different measurements with the corresponding SEM. Control: cells without T-2 toxin exposure; T100: 100 nM, T500: 500 nM, T1000: 1000 nM T-2 toxin treatment.

**Supplementary Table 2.** Significantly different metabolites between the control group and cell-free medium. Statistics were calculated by ANOVA, pairwise comparisons by Tukey's HSD. * p < 0.05, ** p < 0.01, *** < 0.001.

**Supplementary Table 3.** Concentration of the different metabolites in the treatment groups measured by AbsoluteIDQ p180 kit. Control: cells without T-2 toxin exposure; T100: 100 nM, T500: 500 nM, T1000: 1000 nM T-2 toxin treatment.
